# In Their Own Words: The Significance of Participant Perceptions in Assessing Entomology Citizen Science Learning Outcomes Using a Mixed Methods Approach

**DOI:** 10.3390/insects9010016

**Published:** 2018-02-06

**Authors:** Louise I. Lynch, Jenny M. Dauer, Wayne A. Babchuk, Tiffany Heng-Moss, Doug Golick

**Affiliations:** 1 Department of Entomology, University of Nebraska-Lincoln, ENTO 103B, Lincoln, NE 68583, USA; dgolick2@unl.edu; 2 School of Natural Resources, University of Nebraska-Lincoln, HARH 502, Lincoln, NE 68583, USA; jenny.dauer@unl.edu; 3 Department of Educational Psychology, University of Nebraska-Lincoln, TEAC 225, Lincoln, NE 68588, USA; wbabchuk1@unl.edu; 4 College of Agricultural Sciences and Natural Resources, University of Nebraska-Lincoln, AGH 103, Lincoln, NE 68583, USA; thengmoss2@unl.edu

**Keywords:** citizen science, mixed methods, perceived influence, self-efficacy, attitude towards insects

## Abstract

A mixed methods study was used to transcend the traditional pre-, post-test approach of citizen science evaluative research by integrating adults’ test scores with their perceptions. We assessed how contributory entomology citizen science affects participants’ science self-efficacy, self-efficacy for environmental action, nature relatedness and attitude towards insects. Pre- and post-test score analyses from citizen scientists (*n* = 28) and a control group (*n* = 72) were coupled with interviews (*n* = 11) about science experiences and entomological interactions during participation. Considering quantitative data alone, no statistically significant changes were evident in adults following participation in citizen science when compared to the control group. Citizen scientists’ pre-test scores were significantly higher than the control group for self-efficacy for environmental action, nature relatedness and attitude towards insects. Interview data reveal a notable discrepancy between measured and perceived changes. In general, citizen scientists had an existing, long-term affinity for the natural world and perceived increases in their science self-efficacy, self-efficacy for environmental action, nature relatedness and attitude towards insects. Perceived influences may act independently of test scores. Scale instruments may not show impacts with variances in individual’s prior knowledge and experiences. The value of mixed methods on citizen science program evaluation is discussed.

## 1. Introduction

Citizen science programs have increasingly pursued educational goals in addition to research objectives. By engaging the public in authentic research, citizen science is widely championed for its potential to strengthen participants’ understanding of science, environmental learning and critical thinking skills [1,2,3,4]. This capacity has produced a natural synergy with science literacy initiatives [1,5]. Traditionally associated with formal education, the concept of science literacy has expanded to include the general public, individuals of all ages, and informal science education opportunities [6]. This expansion may be connected to the recognition that science is ever evolving, requires a lifelong commitment beyond formal education and that Americans learn most of their science outside of school [7,8]. Science literacy is a complex and multi-faceted construct with numerous definitions and evaluation methods, but typically refers to an individual’s attitude towards science, understanding of science concepts and the process of science, and their ability to employ this understanding in everyday life and decision-making [9,10,11]. The judgment of one’s ability to perform a task within a specific domain, or their self-efficacy, can greatly impact performance, persistence and willingness to try challenging tasks, such as utilizing the scientific process [12]. Self-efficacy positively predicts effort expenditure, persistence and performance [13,14]. Citizen scientist projects that support or increase self-efficacy may encourage participants to persist in understanding of science concepts and the process of science and thus influence their science literacy. While citizen science may expose the public to science concepts and authentic research, limited participation should not be expected to produce experts. However, the *pieces* for improving components of science literacy are present in citizen science projects; these projects have sound strategies using the scientific method, and provide public access to experts with background and procedural knowledge. Citizen science can provide beneficial interactions with professional science and positive interactions with nature, all of which may influence different aspects of an individual’s science literacy.

Hundreds of citizen science programs are available to support informal science learning in a diversity of fields, providing a forum for scientific thinking [15]. Citizen science has been categorized as experiential education [16]. Experiential learning theory conceives learning as a continuous process based in experience [17] and is a prominent learning method in adulthood, operating on the premise that learning is achieved through authentic involvement in an activity [18,19]. Regardless of the field, each citizen science program offers participants exposure to science within a specific domain, from astronomy to entomology to zoology. In principle, the experiential nature of citizen science should positively influence participants’ science literacy. Citizen science researchers often focus on particular components of science literacy, including knowledge gains, enactment of conservation behaviors, attitudes towards science, and connectivity to nature [16,20,21,22,23,24,25,26]. It is important to demonstrate the impacts of citizen science participation to ensure that educational goals are met, justify time and monetary investments for public participation in research, and build the momentum behind meaningful free-choice learning opportunities.

There is limited research on the influence of citizen science programs on volunteers and results have been, to a certain degree, mixed (Table 1). On the positive side, researchers have documented gains in knowledge about birds [16,22,23] and invasive plants [20,24]. Positive influences on community connectedness, sense of place, and conservation behaviors have been detailed in ornithology [22,23] and stream monitoring [25] projects. In addition, Price and Lee (2013) [26] documented improvements in attitudes towards science and epistemological beliefs about the nature of science for volunteers in an astronomy program. In contrast, some projects have indicated citizen science did not influence volunteers’ attitudes towards science and the environment [16,21] or conservation behavior [21,24]. No changes were detected in science knowledge or understanding of the science process in pollinator [21], stream monitoring [25] and invasive plant monitoring [24] projects.

A potentially useful and underutilized approach in the study of citizen science can be drawn from the contemporary mixed methods movement which has emerged as a powerful approach in the fields of education, social science and health sciences [27,28,29,30,31,32,33]. This methodological approach affords greater insight into a phenomenon by the meaningful integration of both statistical trends (close-ended, quantitative data) and personal experiences (open-ended, qualitative data) thereby achieving deeper insight than would be gathered by either data set alone [28,34]. Mixed methods research is useful when neither quantitative nor qualitative data alone are sufficient to characterize a research problem or question. For example, researchers employing this methodology may first carry out qualitative analysis of interviews to develop an instrument or follow up with interviews to explain unexpected quantitative results, as is the case of this study. The Cornell Lab of Ornithology, a source of seminal assessment research on citizen science, has recognized the potential of mixed methods research for evaluating citizen science impacts [35].

This study focuses on exploring change in four constructs associated with science literacy within the domain of entomology: an individuals’ confidence in their ability to learn about and do science (science self-efficacy), confidence in their ability to address environmental concerns (self-efficacy for environmental action), interest in the natural world (nature relatedness) [36], and attitude towards insects. Regarding the first two constructs, self-efficacy is the judgment of one’s ability to perform a task within a specific domain. It impacts an individual’s motivation to learn [37] and their persistence to try challenging tasks within a specific domain [12,38]. Self-efficacy is improved through constructive feedback on performance, encouragement and modeling [12], all of which citizen science program leaders may provide when reporting results, providing research updates and granting citizen scientists access to project data for personal use and review. Increased self-efficacy should positively predict effort expenditure, persistence and performance [13,14] in behaviors and skills associated with science and pro-environmental behaviors. Thus, we hypothesized that entomology citizen science programs provide opportunities to positively influence participants’ science self-efficacy and self-efficacy for environmental action.

The third and fourth constructs that are the focus of this study are nature relatedness and attitude towards insects. Entomology citizen science programs hold great potential to support critical invertebrate conservation efforts [39] as well as provide an educational opportunity to interact with insects in a positive way. Insects are abundant, easily observable, and found in a variety of habitats, however the general public historically tends to dismiss the value of insects and invertebrates, despite their diverse and significant ecological and economic roles [40,41,42]. In formal educational settings, research suggests that physical interactions with insects can positively influence student and teacher knowledge of and attitudes towards insects and other unpopular animals [43,44,45,46,47]. Learning about nature and the environment can maintain and build an individuals’ connection to nature [36,48,49,50]. Thus, we hypothesized that entomology citizen science programs provide opportunities to positively influence participants’ attitude towards insects and their awareness of the natural world.

The purpose of this mixed methods study was to assess how contributory entomology citizen science affects participants’ science self-efficacy, self-efficacy for environmental action, nature relatedness and attitude towards insects. A mixed methods design was selected to transcend the traditional pre- and post-test approach to citizen science evaluative research and to integrate adults’ test scores with their perceptions during participation in entomology citizen science programs. This study employed an intervention explanatory sequential mixed methods design [28]. Phase one of the design was quasi-experimental. Adults were not randomly assigned to the treatment or control group. Rather, all citizen scientists that joined the entomology citizen science programs were invited to participate in this study if they met certain criteria (described below). Control group participants also had to meet certain criteria (described below). Phase one was followed up by an interview-based qualitative phase. The same individuals were used in both phases.

## 2. Materials and Methods

A quasi-experimental design was utilized with one treatment variable of interest: participation in a contributory entomology citizen science project. In contributory citizen science programs, volunteers are asked to contribute data and specimens [2]. Demographic data were collected, including age, gender, highest level of education, ethnic background, and previous or current work as scientists, researcher or science educator. This phase of the study focused on whether entomology citizen science positively influenced the following four constructs in adult participants:

(1) Science self-efficacy
(2) Self-efficacy for environmental action
(3) Nature relatedness
(4) Attitude towards insects

Cornell University’s DEVISE project is developing scales specifically for citizen science audiences. Although undergoing revision, the three scales used in this study had reasonably sound psychometric qualities: the Science Self-Efficacy scale (8 items, *r* > 0.30, *p* < 0.05; α = 0.93; item load > 0.70) [51], Self-Efficacy for Environmental Action scale (8 items, *r* > 0.49, *p* < 0.001; α = 0.89; item load > 0.70) [52], and a shortened Nature Relatedness scale (7 items, expert review) [53]. The versions used during this study may differ from the current version of these scales. The most recent versions and their evaluations are available through the Cornell Lab of Ornithology’s Citizen Science Central portal. In addition, we utilized an 11-item Attitude Towards Insects test developed by UNL Department of Entomology faculty [54] (Golick, Heng-Moss and Weissling, unpublished). Research participants indicated their level of agreement or disagreement with statements on a five-point Likert-type scale. Several features were built into the test to support data quality. Attention filter questions, in which research participants were directed to select ‘Strongly Disagree’ were added to ensure participants were carefully reading and responding to each question. Any response other than ‘Strongly Disagree’ disqualified the entire test from the final data set. Reverse coded questions, in which a question is posed twice but once with a positive root and once with a negative root, ensured that research participants were providing consistent responses. Lastly a test could not be submitted by a research participant unless all questions were answered. One item from the Science Self-Efficacy scale was replaced with an attention filter. This may have affected the integrity of this scale.

Citizen science programs involved in this study were pre-existing and established projects. They varied by hosting institution, research insect, scientific objectives and methods for studying the research insect (Table 2). However, each contributory program required citizen scientists to monitor a target organism, collect entomological data and make regular submissions to the program leaders.

Training sessions, lasting 45–60 min, were carried out by the authors (L.L. and D.G.) to ensure participants were informed of project backgrounds, objectives, protocols and information about the organism being studied. These meetings also allowed us the opportunity to provision attendees with materials needed to participate, including insect nets, glassine envelopes, beetle traps, and educational information. Training sessions were organized for local chapters of the Backyard Bark Beetles, Firefly Watch, Lost Ladybug Project, Milkweed Watch and the Pieris Project. A training session was not carried out by the authors for the Asian Longhorned Beetle Swimming Pool Survey, a project specific to New York State. Care was taken to provide a similar training experience for citizen scientists however, projects varied in duration of participation and the skills utilized during data collection. Citizen science program participants, from which research participants were recruited, included members of the general public that volunteered to join.

Criterion (purposeful) sampling methods were utilized. Research participants met three inclusion criteria: they were 19 years or older, new to the entomology citizen science program, and had not yet turned in data to the project. The first inclusion criterion focused the data on adult, self-directed learners that shared a voluntary decision to participate. The second ensured that research participants were partaking in a new experience. The third ensured that they completed the minimum requirement as a citizen scientist. Research participants were recruited from the following programs: Milkweed Watch (*n* = 13), Backyard Bark Beetles (*n* = 1), Firefly Watch (*n* = 5), Lost Ladybug Project (*n* = 1), the Pieris Project (*n* = 1), and the Asian Longhorned Beetle Swimming Pool Survey (*n* = 7).

A control group was recruited by Qualtrics Panel. Control group participants were drawn from a pool of Unites States citizens and had to be adults (19 years of age or older, as stipulated by state law) with no previous participation in citizen science. Descriptive statistics were calculated from individuals that completed both pre- and posttests (Table 3). Those citizen scientists that had previous science employment (32.1%) included a field researcher in social sciences, elementary school teacher, soil scientist, psychologist, physician (Doctor of Osteopathic Medicine), professor and several Nebraska Master Naturalists.

The measurement scales were distributed online through Qualtrics^TM^ survey software (Qualtrics, Provo, UT, USA). Hard copies were available for individuals with limited computer or internet access. Measurement scale order was randomized within each pre- and posttest. Research participants completed a pre-test at the beginning of training events. In the case of the Asian Longhorned Beetle Swimming Pool Survey, pre-tests were completed at the beginning of the project season. Posttests were distributed once citizen scientists completed their data entry for the season. This varied from seven days to 2 months. The control group completed a posttest approximately 2 months after completing their pre-test.

Semi-structured interview questions were designed in order to provide an open-ended and non-judgmental setting [55] between the research participants and the author. Interview questions focused on perceived changes in science self-efficacy, self-efficacy for environmental action, nature relatedness and attitudes towards insects due to participation in citizen science. We conducted one-on-one interviews over the telephone. Interviews were recorded and non-verbatim transcripts generated by Rev.com.

Research participants were recruited directly from the treatment group of the quasi-experiment and had to have completed both pre- and posttests. Eleven research participants consented to the qualitative phase. Demographic data reflects the treatment group and is provided in Table 4.

Following interviews, triangulation was employed to confirm and corroborate findings and increase the internal validity of the study (see [56] for a comprehensive discussion of internal validity in qualitative research). We provided rich, thick descriptions, calling directly upon research participants’ words and providing details when describing codes, categories and memos. Secondary data sources were used to validate interview statements and represented participation in citizen science, including photographs, entomology research data reported to citizen science projects, blog and Facebook posts, lesson plans, educational brochures, etc. Secondary data sources for each participant ranged from 2 to 10, with a sum of 48 documents.

Data analysis was facilitated with qualitative analysis tools available in MAXQDA 12 (VERBI Software-Consult-Sozialforschung GmbH, Berlin, Germany). Interview analysis consisted of cycles of coding and memoing. Qualitative analysis was done blinded from the research participants’ quantitative test scores to permit an exclusive interpretation of their perceptions before integration. Structural coding [57] was carried out to label broad statements relating to research participants’ science self-efficacy, self-efficacy for environmental action, nature relatedness and attitudes towards insects. Magnitude coding [57] was employed in which interview statements were assigned a symbolic code (minus sign, zero or plus sign) to represent an individual’s self-reported pre- to post-project rating for each construct. Individual’s codes and experiences were compared through memoing, which is the thorough explanation, comparison and organization of code relationships through writing. Once early memos were completed, advanced memos synthesized common themes across research participants’ experiences, allowing themes to emerge.

Integration is a mixture of the quantitative results and qualitative findings for an integrated analysis. This phase of the study focused on how the qualitative findings explain citizen scientists’ change scores in science self-efficacy, self-efficacy for environmental action, nature relatedness and attitudes towards insects.

Pre-test and change scores framed data analysis during integration. Research participants’ memos were sorted by their average pre-test score and change score direction (negative, no change, or positive) within each construct. When grouped by pre-test score, score ranges were composed by individuals of similar levels of self-efficacy, nature relatedness and attitude towards insects. When grouped by change score direction, individuals that numerically moved in the same direction were grouped together. Individuals within each group were compared with each other and compared against other groups.

All recruitment, informed consent and resulting data collection and analysis activities were IRB approved (Approval # 20150615347 EX). Recruitment was carried out at training events and no data was collected outside of the consent process. However, participation in this research was not a requirement to participate in citizen science programs. Prior to interviews, research participants were provided with a sample of the interview questions to facilitate their comfort. Each individual was assigned an alias and identification number.

### Limitations

The authors recognize that the small sample size of the treatment group limited the generalizability of statistical results and acknowledge that a larger sample may have uncovered statistically significant changes in the four constructs studied. The sample size, though small, met the minimum criteria for the scales employed.

## 3. Results

Samples were not normally distributed nor was homogeneity of variance present. Thus, Mann–Whitney *U* tests were conducted to evaluate the hypotheses that citizen science positively influences science self-efficacy, self-efficacy for environmental action, nature relatedness and attitude towards insects in adult participants. For each of the four five-point measurement scales, average change scores for the treatment and control groups were calculated by subtracting pre-test scores from post-test scores. Pre-test scores were also compared.

### 3.1. Science Self-Efficacy

On average, pre-test scores indicated that citizen scientists began their entomology program with higher science self-efficacy (*M* = 4.06, *SD* = 0.57) compared to the control group (*M* = 3.82, *SD* = 0.76). However, this difference was not statistically significant (*z* = −1.09, *p* = 0.28). Both groups remained at approximately the same level and differences between change scores were also statistically insignificant.

Overall, eight of the eleven interviewees expressed confidence in their ability to learn about and do science. High pre- and post-test scores in the treatment group were evident for those individuals expressing pre-existing, long-term interest in science, previous coursework, and participation in additional science-related educational activities. Nance declared, “I do experiments all the time … I think doing experiments just comes naturally.” Vicki, who shares citizen science with her children, mirrored this sentiment: “I have always felt pretty comfortable with science.” Several individuals valued citizen science as an outlet for their existing interest in science. Some individuals explicitly connected their strong science self-efficacy to having advanced degrees in science, including agronomy, biology and elementary science education. On the other hand, Joann presented an interesting case. Despite her years of involvement in ornithology citizen science and recent pursuits in entomology citizen science, she declared, “I’m not too good at science.” She identified many of her friends as bird experts for providing her with the support she needed to participate in citizen science.

Although no statistically significant change occurred in the treatment group, it was evident that most research participants perceived citizen science as having a positive influence on their science self-efficacy (Table 5). Sometimes identifying themselves as “non-academic,” research participants often attributed their strengthened science self-efficacy to the access provided to authentic, academic research by citizen science. Taylor explained, “You don’t have to have an advanced degree in something to participate or pick up or help out...” Vicki explained that being recruited as a citizen scientist bolstered her self-efficacy. “Seeing [researchers] reaching out into the community … sort of gave me more confidence. Seeing that you guys, who are in the field and … have much more knowledge, would reach to us makes it feel like [science] is all more accessible.” Joann indicated increased self-efficacy in her bird identification skills. On the other hand, Barbara felt there was no change, but explicitly attributed this to her science background. Barbara explained, “I have a degree in agronomy. I’ve been doing science for a long time.” Interestingly, even individuals with negative change scores (Bethany, Joann, Sean, Vicki) described increased confidence in science self-efficacy.

### 3.2. Self-Efficacy for Environmental Action

Pre-test scores indicated that citizen scientists began their entomology program with a significantly higher self-efficacy for environmental action (*M* = 4.25, *SD* = 0.47, *z* = −2.52, *p* = 0.01) than the control group (*M* = 3.91, *SD* = 0.66). However, both groups remained at approximately the same level and change score differences were statistically insignificant.

Established pro-environmental behaviors were associated with high pre- and post-test scores in the treatment group. Every interviewee explicitly described existing behaviors to which they have been committed over the past several years, including reducing pesticide use, bicycling instead of driving a car, recycling, incorporating native plants into home landscapes, teaching students about conservation, and encouraging congregation members to be stewards of nature. Sophie provided a particularly exemplary case of pre-existing environmental action: as a young girl growing up on a farm, she asserted that a paint factory upstream was polluting a stream on her family’s property. Despite being hushed by her family, she convinced a state ranger to visit and take water samples.

Although no statistically significant change occurred, nine of the eleven research participants perceived a positive influence on self-efficacy for environmental action (Table 6). Implicit to their discussions was the concept that academic research itself is a way of helping the environment. Thus, by contributing data to a citizen science program and assisting research that aimed to understand insect population declines, they were enacting a pro-environmental behavior. Vicki expressed, “The idea that there are new horizons that have not been touched yet definitely opened up my eyes to feeling that there is a place for me and for my children to have an impact.” Joann intimated that even though a citizen scientist’s individual contribution might be minimal, “… it gives you some kind of feeling that you can contribute … to a project that’s trying to preserve a species. It gives you a feeling that there is something you can do.” Constance further explained that even though she is not “paying a tuition or going to get [an entomology] degree,” citizen science validates her efforts in environmental action. This sentiment was strong among all the citizen scientists that perceived a positive impact on their self-efficacy for environmental action. Taylor and Joann did not perceive changes in this construct, but for different reasons. Taylor did not recall the specific conservation actions presented during his training session. On the other hand, Joann recognized that she was already very active and confident in her ability to impact the environment by providing insect and bird-friendly habitat on her property.

### 3.3. Nature Relatedness

Pre-test scores indicated that citizen scientists began their entomology program with a significantly higher level of nature relatedness (*M* = 4.38, *SD* = 0.45, *z* = −2.44, *p* = 0.02) compared to the control group (*M* = 3.91, *SD* = 0.86). Both groups remained at approximately the same level and change score differences were statistically insignificant. Table 7 shows these results along with abbreviated qualitative findings.

All interviewees, barring one, indicated a strong, pre-existing interest in the natural world. This affinity provided context for the treatment group’s high pre- and post-test scores. Participants’ interest was typically connected to early childhood memories growing up in, and regularly interacting with, a rural or suburban environment. Barbara’s sentiment, “I spent most of my childhood outside, all the time,” reflects similar recollections from Constance, Joann, Nance, Sophie, and Vicki. Some interviewees attributed their pre-existing interest to personal domains other than their childhood. Tom connected his interest in the natural world to his hunting and fishing hobbies. Sean’s academic science background, coupled with wanting to encourage his son’s connection to nature, was the root of his nature relatedness. Brent associated his standing interest in nature to his career as a general construction lobbyist, for which he regularly discusses environmental conservation issues with colleagues as well as his children.

Interviewees were evenly split when describing the impact of citizen science on their awareness and interest in the natural world. Even individuals that exhibited no change in test scores each described a perceived increase in awareness of insect diversity. Bethany, Constance, Joann, Nance, Sean, and Vicki all indicated that citizen science positively impacted their nature relatedness. They linked this change to their increased awareness of the program’s focal insect, whether it was milkweed beetles (*Tetraopes* spp., F. Cerambycidae, O. Coleoptera), fireflies (F. Lampyridae, O. Coleoptera) or lady beetles (F. Coccinellidae, O. Coleoptera). Despite feeling a strong connection to nature, Constance asserted the positive influence of citizen science on her nature relatedness: “I don’t know how it couldn’t but help.” Vicki credited her children’s wonder at nature to opening her own eyes “to those things that you maybe start to glaze over as you get older.” Sean also described his citizen science experience as a spark to reconnect, “I just use it to motivate me to get out and be outside in nature again.” On the other hand, Barbara, Brent, Sophie, and Tom maintained that citizen science did not necessarily change their awareness and interest in the natural world, rather provided them with an outlet. Barbara explained, “I wouldn’t say [citizen science has changed my nature relatedness] in a larger sense … because I’ve always been that way. I think just in a small sense, I’m much more aware now of ladybugs.” Sophie elaborated that citizen science helps her preserve her connection to nature: “I was so grateful in my new state … I wondered ‘I’m now in Nebraska. Am I going to be able to continue all the nature … and projects? … [Citizen science] helps me think I can be of value here in Nebraska.”

### 3.4. Attitude towards Insects

Pre-test scores indicated that citizen scientists began their entomology program with a significantly more positive attitude towards insects (*M* = 3.99, *SD* = 0.51, *z* = −4.89, *p* = 0.00) than the control group (*M* = 3.21, *SD* = 0.71). Although both groups exhibited slightly positive change scores, differences were statistically insignificant. Table 8 shows these results along with abbreviated qualitative findings.

Pre-existing respect and positive attitudes towards insects provided context for the high pre- and posttest scores. Every interviewee declared that they have always liked insects to one degree or another. Some participants again linked early childhood experiences to their existing appreciation of insects. Constance’s “old interest” in insects began with monarch butterflies (*Danaus plexippus*, F. Nymphalidae, O. Lepidoptera) when she was 11 years old and has expanded to other insects. Barbara asserted, “from childhood, I’ve always been fascinated by fireflies.” Joann recalled an amusing interaction with her grandparents: “[They] used to have a couple [boxelder bugs] that lived in their house. They had them like pets.” Tom reminisced: “When the kids were real young, we always looked for bugs and insects… It was something we always did.”

Although no statistically significant change was detected in the treatment group’s attitude towards insects, qualitative research uncovered a perceived impact evident in six of the interviewees. They attributed their more positive attitude to the new knowledge they gained about the citizen science program’s focal insect, as well as their more frequent interactions with the insects. Barbara felt that photographing lady beetles (F. Coccinellidae, O. Coleoptera) for the Lost Ladybug Project made her more sympathetic to insects as individuals and had increased her concern for their individual survival and conservation. In several cases, participants associated an increased awareness of insects with an improved attitude towards them. Nance explained that, during participation in Milkweed Watch, “I didn’t realize that there were so many different kinds of milkweed beetle. That was a shock. I didn’t know there were so many different species of milkweed either.” Vicki attributed her participation in Firefly Watch to her newfound aversion to killing insects inside her home. This was confirmed with an item on the test that specifically represents this behavior. Vicki added that her daughter was once again excited about becoming an entomologist after seeing a female entomologist co-lead the Firefly Watch gathering. Brent, Sophie and Tom did not feel their projects had impacted their attitudes and explained they already had positive attitudes and respect for insects.

## 4. Discussion

By employing a mixed methods design, the measured and perceived influences of entomology citizen science participation on science self-efficacy, self-efficacy for environmental action, nature relatedness and attitudes towards insects were probed. Considering the quantitative results alone, one conclusion might be that the tests were not sensitive enough for a science-savvy adult audience. In this study, citizen scientists’ pre-test scores were already high on the five-point scale. Differences in pre-test and change scores could not be explained through interview data. Citizen science program impacts may be unique to each participant; thus, a traditional scale instrument may not show impacts with the variances present due to the prior knowledge and experiences of each individual. Although the scales may not have detected changes in citizen scientists, three of the scales did distinguish citizen scientists from non-citizen scientists. Even though the control group had slightly positive mean pre-test scores, save attitude towards insects, their average scores were significantly lower than citizen scientists. Adult citizen scientists in our programs “stacked the deck” with their strong science background, pre-existing high levels of science self-efficacy, self-efficacy for environmental action, nature relatedness and attitude towards insects. This phenomenon may affect other researchers attempting to capture change in their project participants. The authors of the DEVISE scales point out that maintaining high self-efficacy levels in individuals should be considered a positive project outcome. Our results support this advice and extend it to attitudes towards insects.

In this study, the authors recognize that the small sample size of the treatment group limited the generalizability of statistical results and acknowledge that a larger sample may have uncovered significant changes in the four constructs studied. However, the small sample size reflects the reality and difficulty of getting citizen scientists to not only volunteer their time for a program, but for educational research as well. A mixed methods approach was invaluable for providing guidelines for comprehensive analysis and building on the survey results that *were* available. In addition, a mixed methods approach uncovered unique, situational impacts on participants in addition to survey results. For example, Bethany reported, “since [the Firefly Watch gathering], I have always picked [insects] up on a piece of paper and threw them outside.” Her behavior change would have been missed through surveys alone. Considering the quantitative and qualitative results *together*, the rich benefit of qualitative data and mixed methods research becomes evident. In our study, a notable discrepancy was uncovered between measured and perceived changes; several citizen scientists *perceived* increases in their science self-efficacy, self-efficacy for environmental action, nature relatedness and attitude towards insects, while the quantitative derived data did not find these results. Similar findings have been reported using mixed methods approaches in other studies [58]. Several research participants suggested the invitation to participate in authentic research through citizen science increased their science self-efficacy and self-efficacy for environmental action. Perceptions acted independently of the scales utilized in this study and may have important long-term implications. Positive perceptions may encourage citizen scientists to persist in a project or join others. Sustained engagement in citizen science programs may provide the prolonged, authentic, learning commitment needed to positively influence science literacy components over time. Perceived positive program impacts may encourage citizen scientists to further explore a research interest or problem and share their science knowledge and experiences with people around them. This may support an individual’s potential to influence educational, behavioral or attitude changes in people within their social circle. Each entomology citizen science program varied in research objectives (pest monitoring, genetic research, conservation and monitoring) and data requests (insect specimens, observational data, photographs, etc.). This may have impacted statistical results in participants’ science self-efficacy, self-efficacy for environmental action, nature relatedness and attitude towards insects. However, follow-up interviews illuminated similarities, rather than differences, that transcended project differences.

While test scores suggest that there was no statistical impact on participants, interview comments revealed that participation in entomology citizen science provided them an opportunity to pursue and cultivate their interests and science knowledge. Even though citizen scientists’ contributory involvement was limited to specimen and data collection and submission, interviews made it clear that their involvement was personally valuable and provided a forum to discuss relevant science and conservation topics within the field of entomology. Self-efficacy is, by definition, an individual’s perception of their ability within a specific domain [38]. Within this study, high levels of science self-efficacy and self-efficacy for environmental action were statistically sustained and perceived as either improving or being maintained. This suggests that entomology citizen science can provide a supportive science experience for adults and that actual impacts may act independently of measured results.

## 5. Conclusions

Mixed methods research provides the citizen science community with an avenue to generate deeper understanding of program impacts and may prevent novel outcomes from going unnoticed. Mixed methods may help program evaluators better acknowledge the diverse audiences that make up citizen scientists, including science-engaged adults and even career scientists. The authors strongly encourage citizen science program evaluators to pursue mixed methods research to evaluate program impacts, including follow-up interviews, or even open-ended questionnaires. These research strategies will offer contextual data that is disregarded by quantitative methods alone. They offer opportunity to uncover perceived impacts, describe discrepancies, explain unexpected quantitative results, and even discover new measurable impacts. A variety of mixed methods approaches are available to integrate detailed perspectives, rich descriptions and contextual insights of citizen science with statistical findings [28], whether significant or not.

This study focused on adult participants that voluntarily participated in contributory entomology citizen science. Future studies may explore discrepancies between research participants’ perceived influences and statistical scores and assist in the development of new scales for measuring various components of science literacy. Discrepancies, or congruity, between perceived and measured impacts also may vary in different age groups, science fields, formal versus informal educational settings and cultures. Understanding the influence of contextual variety would allow the citizen science community to better develop assessment strategies and focus learning objectives.

## Figures and Tables

**Table 1:** Quantitative (quant.) and mixed (quantitative and qualitative) data analysis approaches and project outcomes for various citizen science evaluation studies.

Authors	Data Analysis	Project Field	No Change Detected	Increase Detected
Overdevest et al. (2004) [25]	Quant.	Stream monitoring	Knowledge	Political action Community connectedness
Brossard et al. (2005) [16]	Quant.	Ornithology	Attitude toward science Environment attitude Scientific process	Knowledge
Jordan et al. (2011) [24]	Quant.	Invasive plant monitoring	Scientific process Behavior change	Knowledge
Druschke and Seltzer (2012) [21]	Quant.	Entomology	Knowledge Conservation behavior Attitude towards science	
Evans et al. (2005) [22]	Mixed	Ornithology		Knowledge Behavior Sense of place
Cronje et al. (2011) [20]	Mixed	Invasive plant monitoring		Knowledge
Price and Lee (2013) [26]	Mixed	Astronomy		Attitude towards science Epistemological beliefs about nature of science
Haywood et al. (2016) [23]	Mixed	Coastal monitoring/Ornithology		Sense of place Knowledge and skills Conservation action

**Table 2:** Citizen science program characteristics including project name, Order (O.) and Family (F.) of research insect, research objectives, data collected by citizen scientists and the number of research participants from each project (sample size).

Project Name	Research Insect	Research Objectives	Data Collected	Sample Size
Asian Longhorned Beetle Swimming Pool Survey	*Anoplophora glabripennis* (O. Coleoptera, F. Cerambycidae)	Pest monitoring	Insect specimens	7
Backyard Bark Beetles	Subfamilies Scolytinae and Platypodinae (O. Coleoptera, F. Curculionidae)	Pest monitoring	Insect specimens	1
Firefly Watch	O. Coleoptera, F. Lampyridae,	Monitoring/Conservation	Observation data	5
Lost Ladybug Project	O. Coleoptera, F. Coccinellidae	Monitoring/Conservation	Photographs	1
Milkweed Watch	Invertebrates on *Asclepias* species (F. Apocynaceae)	Monitoring/Conservation	Photographs	13
The Pieris Project	*Pieris rapae* (F. Pieridae, O. Lepidoptera)	Genetic research	Insect specimens	1

**Table 3:** Demographic data of treatment and control group participants.

Demographic Attribute	Category	Citizen Scientist (*n* = 28)	Control (*n* = 72)
Number of citizen science projects in which individual participates (%)	1 project 2 projects 3 projects 4 projects	64.3 17.9 14.3 3.4	0.0 0.0 0.0 0.0
Age (Years)	Range Average	28–74 55.6	20–81 39.3
Sex (%)	Female Male	50.0 50.0	57.0 43.0
Highest level of formal education (%)	HS/GED Some college Associate’s Degree Bachelor’s Degree Master’s Degree Doctoral Degree	0.0 7.1 7.1 46.4 21.4 17.9	16.7 36.1 4.2 26.4 12.5 4.2
Previous employment as scientist, researcher or science educator (%)	Yes No	32.1 67.9	12.5 87.5
Ethnic background	Caucasian	96.4	68.1

**Table 4:** Demographic data of interviewees.

Demographic Attribute	Category	Interviewees (*n* = 11)
Number of citizen science projects in which individual participates	1 project 2 projects 3 projects 4 projects	8 1 1 1
Age (years)	Range Mean	33–67 52
Sex (*n*)	Female Male	7 4
Highest level of formal education (*n*)	Some college Bachelor’s degree Master’s degree Doctoral degree	1 5 3 2
Previous employment as scientist, researcher or science educator (*n*)	Yes No	3 8
Ethnic background (*n*)	Caucasian	11
Aliases	Barbara, Bethany, Brent, Taylor, Constance, Joann, Nance, Sean, Sophie, Tom, Vicki	
Citizen science projects represented in interview pool	Asian Longhorned Beetle Swimming Pool Survey, Backyard Bark Beetles, Firefly Watch, The Lost Ladybug Project, Milkweed Watch, The Pieris Project	

**Table 5:** Joint display of science self-efficacy mean (M) pre-test and change scores, standard deviations (SD), Mann–Whitney U *p*-scores and qualitative findings.

Science Self-Efficacy	*p* Value	Citizen Scientists (*n* = 28)		Control (*n* = 72)	
		M	SD	M	SD
Pre-test score	0.28	4.06	0.57	3.82	0.76
Change score	0.15	−0.01	0.26	−0.13	0.54
Qualitative findings	Pre-existing confidence in ability to learn about and do science; Perceived positive change in science-self efficacy attributed to participation in authentic research				

**Table 6:** Joint display of self-efficacy for environmental action mean (M) pre-test and change scores, standard deviations (SD), Mann–Whitney U *p*-scores (* significant at *p* < 0.05) and qualitative findings.

Self-Efficacy for Environmental Action	*p* Value	Citizen Scientists (*n* = 28)		Control (*n* = 72)	
		M	SD	M	SD
Pre-test score	0.01 *	4.25	0.47	3.91	0.66
Change score	0.45	−0.02	0.45	−0.12	0.51
Qualitative findings	Established pro-environmental behaviors;				
	Perceived positive change in self-efficacy for environmental action attributed to the act of contributing data to conservation research				

**Table 7:** Joint display of nature relatedness mean (M) pre-test and change scores, standard deviations (SD), Mann–Whitney U *p*-scores (* significant at *p* < 0.05) and qualitative findings.

Nature Relatedness	*p* Value	Citizen Scientists (*n* = 28)		Control (*n* = 72)	
		M	SD	M	SD
Pre-test score	0.02 *	4.38	0.45	3.91	0.86
Change score	0.42	0.07	0.28	−0.14	0.54
Qualitative findings	High, pre-existing awareness of natural world;				
	Relationship with nature often attributed to early, childhood memories exploring the outdoors; Perceived positive change linked to increased awareness of project’s focal insect(s)				

**Table 8:** Joint display of attitude towards insects mean (M) pre-test and change scores, standard deviations (SD), Mann–Whitney U *p*-scores (* significant at *p* < 0.05) and qualitative findings.

Attitude towards Insects	*p* Value	Citizen Scientists (*n* = 28)		Control (*n* = 72)	
		M	SD	M	SD
Pre-test score	0.00 *	3.99	0.51	3.21	0.71
Change score	0.72	0.00	0.32	−0.03	0.54
Qualitative findings	Positive, pre-existing attitude towards insects;				
	Perceived positive change linked to increased knowledge of species diversity, interactions with new insect species and newly developed aversion to killing insects				
